# Efficacy and Safety of GLP‐1 and Dual GIP/GLP‐1 Receptor Agonists in Idiopathic Intracranial Hypertension: A Systematic Review and Meta‐Analysis

**DOI:** 10.1111/ene.70358

**Published:** 2025-09-12

**Authors:** Maria‐Ioanna Stefanou, Irini Chatziralli, Vaia Lambadiari, Annerose Mengel, Aikaterini Foska, Maria Chondrogianni, Eleni Bakola, Panagiota‐Eleni Tsalouchidou, Dimos D. Mitsikostas, Gerasimos Siasos, Ulf Ziemann, Georgios Tsivgoulis

**Affiliations:** ^1^ Second Department of Neurology “Attikon” University Hospital, School of Medicine, National and Kapodistrian University of Athens Athens Greece; ^2^ Department of Neurology & Stroke Eberhard‐Karls University of Tübingen Tübingen Germany; ^3^ Second Department of Ophthalmology Attikon University Hospital, National and Kapodistrian University of Athens Athens Greece; ^4^ Second Department of Internal Medicine “Attikon” University Hospital, School of Medicine, National and Kapodistrian University of Athens Athens Greece; ^5^ Hertie Institute for Clinical Brain Research Eberhard‐Karls University of Tübingen Tübingen Germany; ^6^ First Neurology Department Aeginition Hospital, Medical School, National and Kapodistrian University of Athens Athens Greece; ^7^ Third Department of Cardiology Sotiria Thoracic Diseases General Hospital, National and Kapodistrian University of Athens Athens Greece

**Keywords:** exenatide, GIP/GLP‐1, GLP‐1, headache, intracranial hypertension, tirzepatide

## Abstract

**Background:**

Repurposing glucagon‐like peptide‐1 (GLP‐1) and GIP/GLP‐1 receptor agonists (RAs) for idiopathic intracranial hypertension (IIH) represents an attractive alternative to current treatments, supported by evidence of potent metabolic effects and reductions in cerebrospinal fluid secretion and intracranial pressure in vivo.

**Methods:**

We evaluated the safety and efficacy of GLP‐1 RAs and GIP/GLP‐1 RAs in IIH. MEDLINE and Scopus databases were searched for randomized‐controlled trials (RCT), non‐randomized clinical trials, or registries in adults with IIH.

**Results:**

Two clinical trials (one RCT and one non‐randomized case–control) and two registries, comprising 1550 IIH patients (768 receiving GLP‐1 or GIP/GLP‐1 RAs) were included. Compared with standard‐of‐care, GLP‐1 or GIP/GLP‐1 RAs were associated with a significantly lower risk of papilledema (RR: 0.25; 95% CI: 0.15–0.43; *p* < 0.01) and visual disturbances or blindness (RR: 0.41; 95% CI: 0.18–0.92; *p* = 0.03), and a near‐significant trend toward reduced headache risk (RR: 0.61; 95% CI: 0.34–1.07; *p* = 0.08). Additionally, GLP‐1 RAs significantly reduced monthly headache days at 3 months (MD = −3.64; 95% CI: −6.26 to −1.03; *p* < 0.01) and at the end of follow‐up (MD = −4.82; 95% CI: −8.80 to −0.85; *p* = 0.02). No association was detected between GLP‐1 RAs and body mass index. No serious adverse events or treatment discontinuations were reported; mild gastrointestinal adverse events and nausea occurred in 88% (95% CI: 0.46–1.00) of GLP‐1 RA‐treated patients.

**Conclusions:**

GLP‐1 and dual GIP/GLP‐1 RAs are associated with a significantly lower risk of papilledema and visual disturbances or blindness and a lower headache risk compared with standard‐of‐care. Additionally, GLP‐1 RAs significantly reduce the monthly headache burden. Well‐designed RCTs are needed to robustly evaluate the effects of GLP‐1 and GIP/GLP‐1 RAs in IIH, which likely extend beyond their weight‐loss‐inducing properties.

**Trial Registration:**

PROSPERO registration ID: CRD42025650082

## Graphical abstract

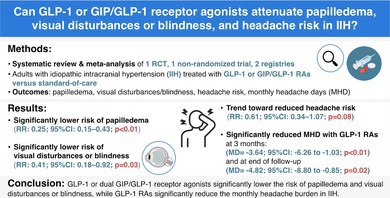

## 1 Introduction

Recent epidemiological trends indicate a significant rise in the prevalence and incidence of idiopathic intracranial hypertension (IIH) in Western countries. Reports from the United States and the United Kingdom indicate a 1.5‐ to 3‐fold increase in both prevalence and incidence over the past decade, with prevalence estimates ranging from 10 to 76 cases per 100,000 population and incidence rates from 2 to 7.8 cases per 100,000 annually [1, 2, 3, 4]. These epidemiological figures, alongside the incremental healthcare burden of IIH, closely parallel the global rise in obesity [4].

While the pathophysiological mechanisms implicated in IIH remain to date partly elucidated, obesity is recognized as causally correlated and the only identified modifiable risk factor [5]. IIH predominantly affects women of reproductive age, with over 90% of affected individuals classified as overweight or obese [6]. Weight management strategies comprise the cornerstone of therapeutic approaches, with weight reduction—typically in the range of 10% sufficing for IIH remission; yet, fewer than half of patients achieve this target within 1 year through high‐intensity lifestyle interventions [6, 7, 8]. With current pharmacotherapies remaining limited in efficacy, refractory IIH cases, particularly those presenting with debilitating chronic headache, progressive papilledema, or imminent visual loss, frequently require surgical management, including bariatric surgery, cerebrospinal fluid diversion procedures, or optic nerve sheath fenestration [9, 10, 11].

Repurposing glucagon‐like peptide‐1 receptor agonists (GLP‐1 RAs) and dual GIP/GLP‐1 receptor agonists (GIP/GLP‐1 RAs) for IIH represents an attractive alternative to currently available IIH treatments. Initially developed for the treatment of type‐2 diabetes mellitus (T2DM), GLP‐1 and GIP/GLP‐1 RAs are presently licensed as anti‐obesity agents for obese or overweight adults with a body mass index (BMI) ≥ 30 kg/m^2^ or a BMI ≥ 27 kg/m^2^ with at least one cardiovascular risk factor (such as prediabetes, diabetes, hypertension, dyslipidemia, and elevated waist circumference) or obesity‐related comorbidity (including non‐alcoholic fatty liver disease and obstructive sleep apnea) [12]. As incretin‐based therapies, GLP‐1 RAs exert pleiotropic effects, with potential utility in IIH extending beyond weight loss and satiety induction to include (i) reduced cerebrospinal fluid (CSF) secretion in preclinical IIH models via binding to GLP‐1 receptors in the choroid plexus, and (ii) pain relief through antinociceptive effects in the central nervous system [7, 13, 14]. In addition, dual GIP/GLP‐1 RAs, also classified as incretin‐based therapies, act through synergistic binding to peripheral and central GIP and GLP‐1 receptors, demonstrating superior glucose‐regulating properties and inducing more potent, sustained weight loss compared with single GLP‐1 RAs [15].

Given the compelling preclinical data and the expanding applications of GLP‐1 and GIP/GLP‐1 RAs, their use in IIH has already been incorporated into clinical practice guidelines, though evidence from dedicated randomized‐controlled clinical trials (RCTs) is limited [16]. To this end, the aim of the present systematic review and meta‐analysis is to comprehensively evaluate the so‐far available clinical data on the safety and efficacy of GLP‐1 and GIP/GLP‐1 RAs in IIH.

## 2 Methods

### 2.1 Standard Protocol Approvals and Registrations

Reporting adheres to the Preferred Reporting Items for Systematic Reviews and Meta‐Analyses (PRISMA) statement [17]. As per study design (systematic review and meta‐analysis) no Ethical Committee approval was required. The study protocol, comprising pre‐determined PICOS (Population, Intervention, Comparison, Outcome and Study) framework, was a priori designed and registered at the PROSPERO database (CRD42025650082).

### 2.2 Data Sources and Searches

Two independent reviewers (MIS, IC) searched for published randomized‐controlled clinical trials (RCTs), matched non‐randomized clinical trials or registries on GLP‐1 or GIP/GLP‐1 RAs in adults with IIH. Eligible studies were identified by systematic search in MEDLINE (via PubMed) and Scopus databases. The combination of search strings for all database queries included combined search terms: “GLP‐1 receptor agonist”, “GIP/GLP‐1 receptor agonist,” “semaglutide,” “lixisenatide,” “exenatide,” “albiglutide,” “liraglutide,” “dulaglutide,” or “tirzepatide” and “idiopathic intracranial hypertension” or “pseudotumor cerebri.” The full search algorithms used in MEDLINE and SCOPUS databases are provided in the Supporting Information. Our search was restricted to clinical trials or registries, while no language restrictions were applied. The search spanned from each electronic database's inception to August 12th, 2025. Manual search of bibliographies of articles meeting study inclusion criteria was additionally performed to ensure the comprehensiveness of the literature.

RCTs, matched non‐randomized clinical trials or registries in adults with IIH under treatment with GLP‐1 or GIP/GLP‐1 RAs were eligible for inclusion. Exclusion criteria comprised: (1) preclinical studies; (2) studies not including ascertained IIH cases as per current diagnostic criteria [18]; (3) reported outcomes not aligned with our inclusion criteria; (4) narrative and systematic reviews, case series or case reports, commentaries, preprints or non‐peer‐reviewed studies, and conference abstracts. In case of studies with overlapping data, the most recent study was retained. All retrieved studies were independently assessed by two reviewers (MIS, IC) and disagreements were resolved by consensus after discussion with a third tie‐breaking evaluator (GT).

### 2.3 Quality Control, Bias Assessment and Data Extraction

For relevant domains of each included study, the risk of bias was assessed using the Cochrane Collaboration risk of bias RoB2 tool [19] and the ROBINS‐I tool (“Risk Of Bias In Non‐randomised Studies—of Interventions”) for RCTs and non‐randomized trials or registries [20], respectively. Three independent reviewers (M.I.S., I.C., V.L.) performed quality control and bias assessment, and in case of disagreement consensus after discussion with the corresponding author (GT) was reached. Data including first author name, publication year, study design and duration, patient population, sample size, and outcomes were extracted from individual studies in structured reports.

### 2.4 Outcomes

An aggregate data meta‐analysis was performed including all identified studies. The primary efficacy outcomes were the risk of (i) papilledema; (ii) visual disturbances or blindness; and (iii) headache. Secondary efficacy outcomes comprised changes in (i) monthly headache days (MHD); (ii) cerebrospinal fluid (CSF) opening pressure or intracranial pressure (ICP); (iii) body weight (kg); (iv) body mass index (BMI; calculated using the formula: BMI = weight [kg]/height [m]^2^); (v) visual acuity, assessed by the logarithm of the minimum angle of resolution (logMAR) chart of the most affected eye; (vi) visual field of the most affected eye, measured by perimetric mean deviation (PMD) in decibels (dB) with the Humphrey visual field analyzer; and (vii) papilledema severity, evaluated by optical coherence tomography (OCT) using as a surrogate marker the peripapillary retinal nerve fiber layer (RNFL) thickness of the most affected eye. All primary and secondary efficacy outcomes of interest were assessed at 3 months and/or at the end of follow‐up, as reported in each included study. The primary safety outcomes were (i) the pooled incidence of serious adverse events (SAEs) and (ii) the pooled incidence of adverse events (AEs) that led to premature treatment discontinuation. Secondary safety outcomes comprised (i) the pooled incidence of mild gastrointestinal AEs and (ii) nausea among patients with IIH treated with GLP‐1 or GIP/GLP‐1 RAs.

### 2.5 Statistical Analysis

R–software, version 3.5.0 (packages: meta and metafor), was used for meta‐analysis. For dichotomous outcomes, the inverse variance method was used to calculate pooled risk ratios (RR) with corresponding 95% confidence intervals (CI). Continuous outcomes were assessed by mean difference (MD) and their corresponding 95% CI. For studies reporting continuous outcomes in median values and corresponding interquartile ranges, we estimated the sample mean and standard deviation using the quantile estimation method [21]. For each dichotomous outcome of interest, the pooled incidence with its corresponding 95% CI was calculated after the implementation of the Freeman‐Tukey variance‐stabilizing double arcsine transformation [22]. For continuous outcome measures calculated in dissimilar ways, standardized mean difference (SMD) estimates were calculated as the mean differences divided by the corresponding pooled standard deviations and were subsequently interpreted using estimates proposed by Cohen [23], according to which an SMD of 0.2 represents a small effect, an SMD of 0.5 represents a medium effect, and an SMD of ≥ 0.8 represents a large effect. In the cases where no events were observed for assessed outcomes in included studies, a continuity correction was performed in accordance with the Cochrane Handbook. All estimates were pooled under the random‐effects model (DerSimonian and Laird). Heterogeneity was assessed with the *I* ^2^ and Cochran *Q* statistics. For the qualitative interpretation of heterogeneity, *I* ^2^ values > 50% and values > 75% were considered to represent substantial and considerable heterogeneity, respectively [24]. The significance level was set at 0.1 for the *Q* statistic [25], while the equivalent *z* test with a two‐tailed *p* value < 0.05 was considered statistically significant for each pooled estimate [26].

## 3 Results

### 3.1 Literature Search and Included Studies

The systematic database search yielded a total of 34 and 35 records from the MEDLINE and SCOPUS databases, respectively (Figure S1). After excluding duplicates and initial screening, we retrieved the full text of 18 records that were considered potentially eligible for inclusion. After reading the full‐text articles, 14 were further excluded (Table S1). Finally, we identified 4 eligible studies [7, 27, 28, 29] for inclusion in the systematic review and meta‐analysis, comprising a total of 1550 patients with IIH: 768 in the treatment group, receiving either GLP‐1 RAs (*n* = 575) or GIP/GLP‐1 RAs (*n* = 193) versus 782 in the control group, receiving either the standard of care (*n* = 774) or placebo (*n* = 8) (Table 1).

**TABLE 1:** Main characteristics of studies included in the meta‐analysis.

Study	Year	Medication/type	Study design/country	Population	Study period	Total population (*n*)	Active (*n*)	Placebo or controls (*n*)
Azzam et al.	2025	Tirzepatide SC/dual GIP–GLP‐1 RA	Multicenter, retrospective, propensity score–matched cohort study using TriNetX Global Health Research Network/International (predominantly USA)	Adults ≥ 18 years with IIH diagnosed via ICD‐10, matched for demographics and comorbidities	24 months	386	193	193
Krajnc et al.	2023	Semaglutide (*n* = 11) initiated at 0.25 mg per week and escalated to the maximum tolerated dose or up to 2 mg per week, or liraglutide (*n* = 2) initiated at 0.6 mg per day and escalated to the maximum tolerated dose or up to 3.0 mg per day over 4 weeks SC versus standard of care/GLP‐1 RA	Single‐center, matched case–control trial in which patients who elected to receive GLP‐1 RAs were matched for age, sex, and BMI in a 1:2 ratio to controls receiving standard of care/Austria	Definite IIH according to the modified Friedman criteria, BMI ≥ 30 kg/m^2^, and a follow‐up of ≥ 6 months	24 weeks	39	13	26
Mitchell et al.	2023	Exenatide 10 μg twice daily or placebo SC/GLP‐1 RA	Single‐center, randomized, parallel‐arm, double‐blind, placebo‐controlled trial/United Kingdom	Women aged 18–60 years, who met the diagnostic criteria for IIH. All had normal brain imaging	12 weeks	15	7	8
Sioutas et al.	2025	GLP‐1 receptor agonists (lixisenatide, albiglutide, dulaglutide, semaglutide, liraglutide, and exenatide) SC/GLP‐1 RA	Multicenter, retrospective, propensity score–matched cohort study using TriNetX Global Health Research Network/International (predominantly USA)	Adults ≥ 18 years with IIH diagnosed via ICD‐10, matched for demographics and comorbidities	12 months	1110	555	555

Abbreviations: BMI, body mass index; GLP‐1 RAs, glucagon‐like peptide‐1 receptor agonists; IIH, idiopathic intracranial hypertension; SC, subcutaneous.

### 3.2 Quality Control of Included Studies

The risk of bias assessment of included studies using the Cochrane risk‐of‐bias (RoB 2) tool [19] and the ROBINS‐I tool [20] is presented in Figures S2 and S3. The phase I RCT by Mitchell et al. [7, 27] presented low to moderate risk of bias, while the case–control study by Krajnc et al. [7, 27] and the registry‐based studies by Azzam et al. [29] and Sioutas et al. [28] presented an overall moderate to serious risk of bias as detailed in the Supporting Information.

### 3.3 Quantitative Analyses

**FIGURE 1:**
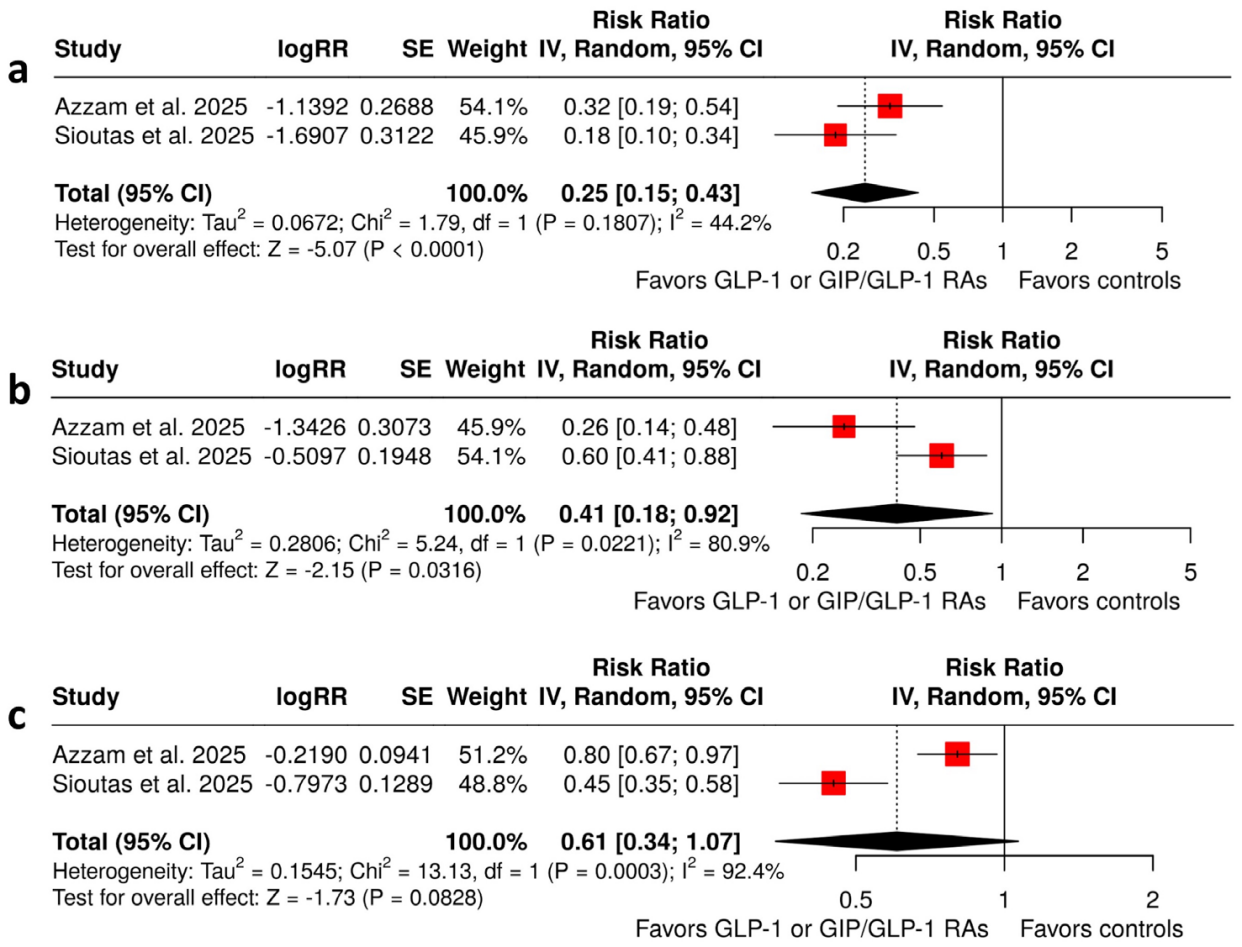
Forest plot comparing the risk of (a) papilledema (b) visual disturbances or blindness, and (c) headache in IIH patients treated with GLP‐1 or GIP/GLP‐1 RAs versus controls.

An overview of the analyses for primary and secondary outcomes is summarized in Table 2. With respect to the primary efficacy outcomes, GLP‐1 or GIP/GLP‐1 RA treatment was associated with a significantly lower risk of papilledema (RR: 0.25; 95% CI: 0.15 to 0.43; *p* < 0.01; 2 studies; *I* ^2^ = 44%; *p* for Cochran's *Q* = 0.18; Figure 1a) and visual disturbances or blindness (RR: 0.41; 95% CI: 0.18 to 0.92; *p* = 0.03; 2 studies; *I* ^2^ = 81%; *p* for Cochran's *Q* = 0.02; Figure 1b) compared with standard of care. In addition, there was a near‐significant trend toward a reduced risk of headache with GLP‐1 or GIP/GLP‐1 RA treatment (RR: 0.61; 95% CI: 0.34 to 1.07; *p* = 0.08; 2 studies; *I* ^2^ = 93%; *p* for Cochran's *Q* < 0.01; Figure 1c).

**TABLE 2:** Overview of the primary and secondary outcomes in patients with idiopathic intracranial hypertension treated with GLP‐1 RAs versus controls.

Clinical outcome	Time point	Statistical measure	Pooled outcome	*p*	Heterogeneity (*I* ^2^, *p* for Cochran *Q*)
Papilledema	End of follow‐up	RR, 95% CI	0.25 (0.15 to 0.43)	**< 0.01**	44%, 0.18
Visual disturbances or blindness	End of follow‐up	RR, 95% CI	0.41 (0.18 to 0.92)	**0.03**	81%, 0.02
Headache	End of follow‐up	RR, 95% CI	0.61 (0.34 to 1.07)	0.08	93%, < 0.01
Monthly headache days	3 months	MD, 95% CI	−3.64 (−6.26 to −1.03)	**< 0.01**	0%, 0.48
	End of follow‐up		−4.82 (−8.80 to −0.85)	**0.02**	0%, 0.68
Body mass index	3 months	MD, 95% CI	−0.07 (−1.05 to −0.90)	0.88	34%, 0.22
	End of follow‐up		−0.57 (−2.62 to 1.48)	0.59	79%; 0.03
Visual acuity, assessed by the logarithm of the minimum angle of resolution (logMAR) chart of the most affected eye	3 months	MD, 95% CI	0.08 (−0.17 to 0.01)	0.07	0%; 0.45
	End of follow‐up		−0.04 (−0.20 to 0.12)	0.63	53%; 0.14
Visual field of the most affected eye, measured by perimetric mean deviation (PMD) in decibels (dB) with the Humphrey visual field analyzer	3 months	SMD, 95% CI	−0.54 (−1.41 to 0.34)	0.23	49%; 0.16
	End of follow‐up		−0.54 (−1.41 to 0.33)	0.23	48%; 0.23
Peripapillary retinal nerve fiber layer (RNFL) thickness of the most affected eye	3 months	SMD, 95% CI	0.33 (−0.39 to 1.04)	0.37	32%; 0.22
	End of follow‐up		0.13 (−0.43 to 0.69)	0.65	0%; 0.52
Pooled incidence of serious adverse events (SAEs) with GLP‐1 RAs	End of follow‐up	Pooled incidence, 95% CI	1% (0 to 0.13)^a^	—	0%; 0.79
Pooled incidence of adverse events (AEs) that led to premature discontinuation of GLP‐1 RAs	End of follow‐up	Pooled incidence, 95% CI	1% (0 to 0.13)^a^	—	0%; 0.79
Pooled incidence of mild gastrointestinal AEs among patients with IIH treated with GLP‐1 RAs	End of follow‐up	Pooled incidence, 95% CI	88% (0.46 to 1.00)	—	71%; 0.06
Pooled incidence of nausea among patients with IIH treated with GLP‐1 RAs	End of follow‐up	Pooled incidence, 95% CI	88% (0.46 to 1.00)	—	71%; 0.06

*Note:* For all outcomes, data from two studies were pooled for each meta‐analysis. Bold indicated the statistically significant *p* values.

Abbreviations: CI, confidence interval; GLP‐1 RAs, glucagon‐like peptide‐1 receptor agonists; MD, mean difference; RR, risk ratio; SMD, standardized mean difference.

^a^ Continuity correction was applied for estimation of the pooled incidence; zero events were recorded for each of the aforementioned safety outcomes in the two included studies.

**FIGURE 2:**
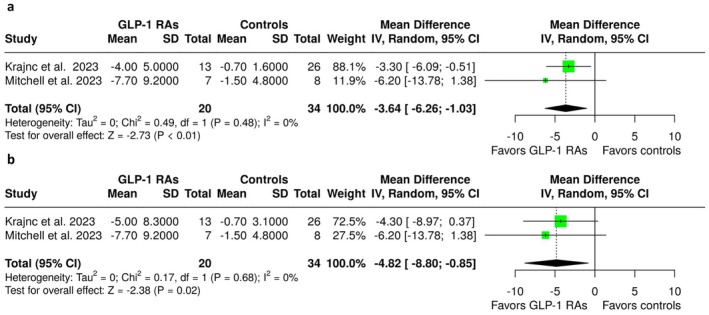
Forest plot comparing the change in monthly headache days in GLP‐1 RA treated IIH patients versus controls (a) at 3 months and (b) at the end of follow‐up.

Regarding secondary efficacy outcomes, treatment with GLP‐1 RAs was associated with a significant reduction in MHD at 3 months (MD = −3.64; 95% CI: −6.26 to −1.03; *p* < 0.01; 2 studies; *I* ^2^ = 0%; *p* for Cochran's *Q* = 0.48; Figure 2a) and at the end of follow‐up (MD = −4.82; 95% CI: −8.80 to −0.85; *p* = 0.02; 2 studies; *I* ^2^ = 0%; *p* for Cochran's *Q* = 0.68; Figure 2b). No association was observed with BMI at 3 months (MD = −0.07; 95% CI: −1.05 to 0.90; *p* = 0.88; 2 studies; *I* ^2^ = 34%; *p* for Cochran's *Q* = 0.22; Figure S4) or the end of follow‐up (MD = −0.57; 95% CI: −2.62 to 1.48; *p* = 0.59; 2 studies; *I* ^2^ = 79%; *p* for Cochran's *Q* = 0.03; Figure S5), though Krajnc et al. [7, 27] reported a significant BMI reduction at 6 months. Pooled analysis for body weight was not possible, with only one study reporting significant reductions at 3 and 6 months [27] compared with usual care. Similarly, data pooling was not possible for CSF opening pressure or changes in ICP, as only one study [7] reported a significant ICP reduction in IIH patients treated with the GLP‐1 RA exenatide versus placebo‐treated controls. This reduction, measured using an implanted telemetric ICP catheter, was evident as early as 2.5 h after GLP‐1 RA administration (with a recorded ICP reduction of −4.2 mmHg, equivalent to −5.7 cm CSF reduction) and persisted at 3 months (with a recorded ICP reduction of −4.1 mmHg, equivalent to −5.6 cm CSF).

**FIGURE 3:**
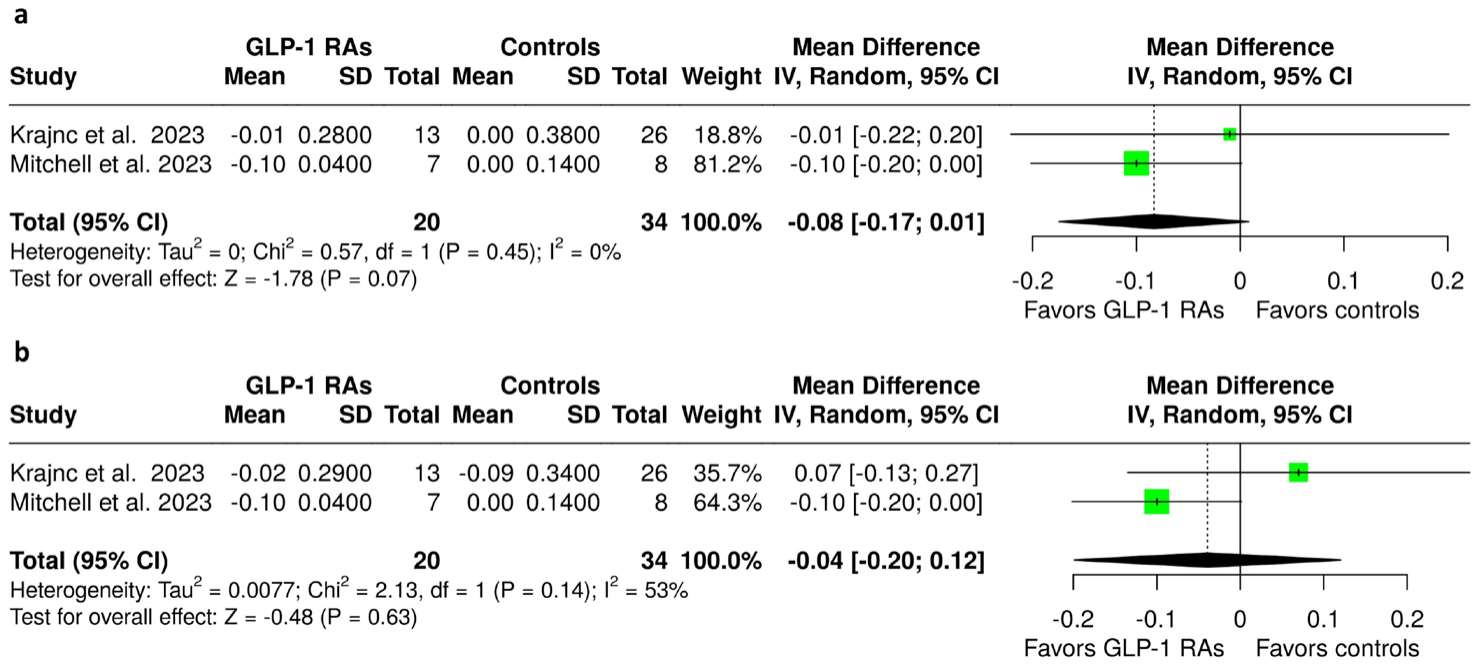
Forest plot comparing the change in visual acuity in GLP‐1 RA treated IIH patients versus controls (a) at 3 months and (b) at the end of follow‐up.

Regarding visual outcomes, a near‐significant trend toward improved visual acuity with GLP‐1 RA treatment was observed at 3 months (MD = −0.08; 95% CI: −0.17 to 0.01; *p* = 0.07; 2 studies; *I* ^2^ = 0%; *p* for Cochran's *Q* = 0.45; Figure 3a); however, this trend did not reach statistical significance at the end of follow‐up (MD = −0.04; 95% CI: −0.20 to 0.12; 2 studies; *p* = 0.63; *I* ^2^ = 53%; *p* for Cochran's *Q* = 0.14; Figure 3b). No associations were observed between GLP‐1 RA treatment and changes in the visual field of the most affected eye at 3 months (SMD = −0.54; 95% CI: −1.41 to 0.34; *p* = 0.23; 2 studies; *I* ^2^ = 49%; *p* for Cochran's *Q* = 0.16; Figure S6) or at the end of follow‐up (SMD = −0.54; 95% CI: −1.41 to 0.33; *p* = 0.23; 2 studies; *I* ^2^ = 48%; *p* for Cochran's *Q* = 0.16; Figure S7). Similarly, no associations were observed for changes in RNFL thickness of the most affected eye at 3 months (SMD = 0.33; 95% CI: −0.39 to 1.04; *p* = 0.37; 2 studies; *I* ^2^ = 32%; *p* for Cochran's *Q* = 0.22; Figure S8) or at the end of follow‐up (SMD = 0.13; 95% CI: −0.43 to 0.69; *p* = 0.65; 2 studies; *I* ^2^ = 0%; *p* for Cochran's *Q* = 0.52; Figure S9).

For safety, the pooled incidence of SAEs among IIH patients treated with GLP‐1 RAs was 1% (95% CI: 0 to 0.13; 2 studies; *I* ^2^ = 0%; *p* for Cochran's *Q* = 0.79; Figure S10) and the pooled incidence of AEs leading to premature discontinuation of GLP‐1 RAs was also 1% (95% CI: 0 to 0.13; 2 studies; *I* ^2^ = 0%; *p* for Cochran's *Q* = 0.79; Figure S11) after continuity correction, with zero events recorded for each of the aforementioned safety outcomes. Among GLP‐1 RA‐treated patients, the pooled incidence of mild gastrointestinal AEs was 88% (95% CI: 0.46 to 1.00; 2 studies; *I* ^2^ = 71%; *p* for Cochran's *Q* = 0.06; Figure S12) and the pooled incidence of nausea was also 88% (95% CI: 0.46 to 1.00; 2 studies; *I* ^2^ = 71%; *p* for Cochran's *Q* = 0.06; Figure S13).

## 4 Discussion

In this systematic review and meta‐analysis, treatment with GLP‐1 or dual GIP/GLP‐1 RAs was associated with a significantly reduced risk of papilledema and visual disturbances or blindness, and with a near‐significant reduction in headache risk compared with standard‐of‐care IIH treatment. By contrast, the secondary efficacy and safety outcomes were derived exclusively from studies of GLP‐1 RAs. GLP‐1 RA treatment was associated with a significant reduction in MHD, with an average decrease of −3.6 and −4.8 days per month in GLP‐1 RA‐treated IIH patients versus controls between baseline and 3 months, and between baseline and the end of follow‐up, respectively. These estimates align with or even marginally exceed the previously established cut‐off of −2 MHD for clinically meaningful treatment benefits in IIH and chronic migraine RCTs [7, 30, 31]. Notably, as indicated in current practice guidelines [10], despite the significant headache morbidity in IIH, no RCT data are available to guide headache management; thus, the current findings advocate for larger, well‐designed trials to robustly evaluate the efficacy of GLP‐1 and dual GIP/GLP‐1 RAs in IIH.

The prior results should also be viewed in conjunction with recently published data from the IIH Pressure Trial (ISRCTN12678718), the double‐blinded, placebo‐controlled phase I trial by Mitchell et al. [7], which assessed the effects of the GLP‐1 RA exenatide on ICP in a cohort of 15 patients with active IIH (7 and 8 of whom received exenatide and placebo, respectively) [32]. In a post hoc exploratory analysis, the authors reported that exenatide was associated with significant improvements in cognitive performance, including enhanced fluid intelligence, processing speed, and episodic memory [33]. Given the growing evidence of positive neurocognitive effects with GLP‐1 RA use, including in IIH cohorts, these agents are increasingly regarded as an attractive alternative to first‐line IIH treatments (e.g., acetazolamide and topiramate), both of which are associated with cognitive side effects and impairment, even in young patients [34, 35].

In parallel with the significant reduction in MHD, a near‐significant trend toward improved visual acuity was detected with GLP‐1 RAs at 3 months, though this trend did not reach statistical significance by the end of follow‐up. No associations were observed between GLP‐1 RAs and changes in visual fields or RNFL thickness of the most affected eye. It should be stressed, however, that short follow‐up periods and sample‐size constraints may have significantly impacted the assessment of visual outcomes. Discordant timing between clinical improvement and changes in neuro‐ophthalmological surrogate markers has frequently been reported in IIH RCTs and real‐world studies, with longitudinal data on Humphrey visual field PMD and OCT measures of RNFL typically demonstrating improvement after a minimum of 12 months of IIH treatment [36, 37]; a fact that may align with the observed reduced risk of papilledema and visual disturbances or blindness with GLP‐1 or GIP/GLP‐1 RA treatment in IIH cohorts with > 12 months of follow‐up [28, 29].

Despite the robust effects of GLP‐1 RAs on body weight and BMI in T2DM and obesity trials [38, 39], our meta‐analysis did not reveal significant associations between GLP‐1 RAs and BMI reduction, although significant heterogeneity between studies was uncovered with the study by Krajnc et al. [7, 27] reporting significant BMI reduction at the end of a 6‐month follow‐up. Data pooling to evaluate effects on body weight was not feasible, as only one of the included studies reported a significant reduction in body weight among IIH patients treated with GLP‐1 RAs (semaglutide or liraglutide) [27]. Prior research in T2DM and overweight/obese patients has demonstrated that the time‐dependent and progressively diverging response curves between GLP‐1 RAs and placebo on metabolic effects, including weight loss, typically attain statistical significance after a minimum of 6 months of treatment [38]. Given that metabolic effects were also secondary outcomes in the included studies, these were likely underpowered to detect metabolic effects, and type II errors may account for the underestimation of GLP‐1 RAs' efficacy in this regard.

Notably, weight‐independent mechanisms may also account for the previous findings. In fact, early effects of GLP‐1 RAs in IIH are unlikely to be related to weight loss, as evidenced by the significant ICP reduction measured via implanted telemetric catheters in IIH patients as early as 2.5 h following exenatide administration [7]. This observation arguably reinforces the preclinical data of reduced CSF production and direct alterations in ICP via GLP‐1 RA‐mediated increase in the intracellular concentration of cyclic adenosine monophosphate and inhibition of the Na+/K+ ATPase pump in the choroid plexus [14, 40].

With respect to safety outcomes, no SAEs or AEs leading to premature discontinuation of GLP‐1 RAs were reported, while the pooled incidence of mild gastrointestinal AEs and nausea was 88% for each outcome among GLP‐1 RA‐treated IIH patients. Although gastrointestinal AEs are typically mild and self‐limited in patients receiving GLP‐1 RA treatment, they may represent a relevant limitation in RCT designs, likely introducing unmasking bias in patients undergoing active treatment [7]. The inclusion of robust physiological primary outcome measures, including optic nerve ultrasound, standardized visual acuity, visual field, and OCT assessments, alongside CSF opening pressure measurements, should thus be considered in future trial designs [41, 42].

Certain limitations of this meta‐analysis should be acknowledged. Two TriNetX registry‐based retrospective studies, one of which evaluated tirzepatide as the only available dataset on a GIP/GLP‐1 RA in IIH to date, alongside one phase I RCT and one non‐randomized matched clinical trial, were included in the pooled analysis [7, 27, 28, 29]. However, the inherent risk of bias, as demonstrated in the quality assessment, limits the generalizability of our findings, which warrant further prospective validation. Given the scarcity of IIH trials, a prior Cochrane review included only two RCTs comparing acetazolamide to placebo [43]. Despite its rising incidence, IIH remains a rare disorder, a fact that explains the disproportionately limited number of dedicated RCTs and the premature termination of the IIH EVOLVE trial (ClinicalTrials.gov ID: NCT05347147), a phase III randomized, placebo‐controlled trial that sought to evaluate presendin, a novel exenatide formulation in IIH patients [44]. This trial was halted prematurely due to slower than expected recruitment, and no active or recruiting RCTs on GLP‐1 RAs in IIH are currently in the pipeline.

To the best of our knowledge, the present meta‐analysis is the first to comprehensively evaluate current evidence on the use of GLP‐1 and dual GIP/GLP‐1 RAs exclusively in IIH. Compared with a previously published systematic review on the role of GLP‐1 RAs for headache and pain disorders, which included fewer studies (*n* = 2), a substantially smaller sample size (*n* = 54 IIH patients), and no GIP/GLP‐1 RA data, the present meta‐analysis provides an updated and more comprehensive synthesis, including pooled quantitative estimates from 1550 IIH patients [40]. With incretin‐based therapies gaining increasing traction, well‐designed real‐world studies are essential to establish their efficacy in IIH. In clinical practice, acknowledgment of IIH as an obesity‐related comorbidity—akin to non‐alcoholic fatty liver disease and obstructive sleep apnea—could also facilitate broader access to these agents [45].

In conclusion, this systematic review and meta‐analysis demonstrates that GLP‐1 or dual GIP/GLP‐1 RAs are associated with a significantly lower risk of papilledema and visual disturbances or blindness compared with the standard of care, while GLP‐1 RAs also significantly reduce the monthly headache burden in IIH. Given the excellent safety profile of these agents and the preliminary efficacy signal, well‐designed and adequately powered trials are needed to determine their role in IIH and expand access to these treatments, particularly in light of their potential to mitigate cognitive side effects of current IIH pharmacotherapies.

## Author Contributions

Conceptualization: G.T.; data curation: M.‐I.S., I.C., V.L., and G.T.; formal analysis: M.‐I.S. and G.T.; investigation: M.‐I.S., I.C., V.L., and G.T.; methodology: M.‐I.S., and G.T.; project administration: G.T.; supervision: G.T.; visualization: M.‐I.S.; writing – original draft: M.‐I.S. and G.T.; writing – review and editing: A.M., A.F., M.C., E.B., P.‐E.T., D.D.M., G.S., and U.Z.

## Ethics Statement

The authors have nothing to report.

## Consent

The authors have nothing to report.

## Conflicts of Interest

The authors declare no conflicts of interest.

## Supporting information


**Data S1:** Supporting Information.

## Data Availability

All data generated or analyzed during this study are included in this article and its Supporting Information files.

## References

[1] J. Chen and M. Wall , “Epidemiology and Risk Factors for Idiopathic Intracranial Hypertension,” International Ophthalmology Clinics 54 (2014): 1–11. 10.1097/IIO.0b013e3182aabf11 PMC3864361 24296367

[2] G. McCluskey , R. Doherty‐Allan , P. McCarron , et al., “Meta‐Analysis and Systematic Review of Population‐Based Epidemiological Studies in Idiopathic Intracranial Hypertension,” European Journal of Neurology 25 (2018): 1218–1227. 29953685 10.1111/ene.13739

[3] L. Miah , H. Strafford , B. Fonferko‐Shadrach , et al., “Incidence, Prevalence, and Health Care Outcomes in Idiopathic Intracranial Hypertension: A Population Study,” Neurology 96 (2021): e1251–e1261. 33472926 10.1212/WNL.0000000000011463 PMC8055349

[4] J. K. Shaia , N. Sharma , M. Kumar , et al., “Changes in Prevalence of Idiopathic Intracranial Hypertension in the United States Between 2015 and 2022, Stratified by Sex, Race, and Ethnicity,” Neurology 102 (2024): e208036. 38181397 10.1212/WNL.0000000000208036 PMC11097766

[5] A. Foska , L. Palaiodimou , M. I. Stefanou , et al., “Telltale Signs of Idiopathic Intracranial Hypertension With Normal Opening Cerebrospinal Fluid Pressure,” Neurohospitalist 13 (2023): 103–106. 36531847 10.1177/19418744221131918 PMC9755610

[6] A. J. Sinclair , M. A. Burdon , P. G. Nightingale , et al., “Low Energy Diet and Intracranial Pressure in Women With Idiopathic Intracranial Hypertension: Prospective Cohort Study,” BMJ 341 (2010): c2701. 20610512 10.1136/bmj.c2701 PMC2898925

[7] J. L. Mitchell , H. S. Lyons , J. K. Walker , et al., “The Effect of GLP‐1RA Exenatide on Idiopathic Intracranial Hypertension: A Randomized Clinical Trial,” Brain 146 (2023): 1821–1830. 36907221 10.1093/brain/awad003 PMC10151178

[8] J. Hoffmann , S. P. Mollan , K. Paemeleire , C. Lampl , R. H. Jensen , and A. J. Sinclair , “European Headache Federation Guideline on Idiopathic Intracranial Hypertension,” Journal of Headache and Pain 19 (2018): 93. 30298346 10.1186/s10194-018-0919-2 PMC6755569

[9] V. Yumuk , C. Tsigos , M. Fried , et al., “European Guidelines for Obesity Management in Adults,” Obesity Facts 8 (2015): 402–424. 26641646 10.1159/000442721 PMC5644856

[10] S. P. Mollan , B. Davies , N. C. Silver , et al., “Idiopathic Intracranial Hypertension: Consensus Guidelines on Management,” Journal of Neurology, Neurosurgery, and Psychiatry 89 (2018): 1088–1100. 29903905 10.1136/jnnp-2017-317440 PMC6166610

[11] A. Örgel , B. Bender , M. I. Stefanou , H. Hurth , T. K. Hauser , and M. Horger , “Image Findings in Spontaneous Intracranial Hypotension (SIH),” Röfo 190 (2018): 219–224. 29495052 10.1055/s-0043-122166

[12] M. I. Stefanou , L. Palaiodimou , A. Theodorou , et al., “Risk of Major Adverse Cardiovascular Events and All‐Cause Mortality Under Treatment With GLP‐1 RAs or the Dual GIP/GLP‐1 Receptor Agonist Tirzepatide in Overweight or Obese Adults Without Diabetes: A Systematic Review and Meta‐Analysis,” Therapeutic Advances in Neurological Disorders 17 (2024): 17562864241281903. 39345822 10.1177/17562864241281903 PMC11437580

[13] E. J. Go , S. M. Hwang , H. Jo , et al., “GLP‐1 and Its Derived Peptides Mediate Pain Relief Through Direct TRPV1 Inhibition Without Affecting Thermoregulation,” Experimental & Molecular Medicine 56 (2024): 2449–2464. 39482537 10.1038/s12276-024-01342-8 PMC11612315

[14] H. F. Botfield , M. S. Uldall , C. S. J. Westgate , et al., “A Glucagon‐Like Peptide‐1 Receptor Agonist Reduces Intracranial Pressure in a Rat Model of Hydrocephalus,” Science Translational Medicine 9 (2017): 9. 10.1126/scitranslmed.aan0972 28835515

[15] T. Salmen , L. I. Serbanoiu , I. C. Bica , et al., “A Critical View Over the Newest Antidiabetic Molecules in Light of Efficacy‐A Systematic Review and Meta‐Analysis,” International Journal of Molecular Sciences 24 (2023): 9760. 37298707 10.3390/ijms24119760 PMC10253587

[16] S. Abbott , F. Chan , A. A. Tahrani , et al., “Weight Management Interventions for Adults With Idiopathic Intracranial Hypertension: A Systematic Review and Practice Recommendations,” Neurology 101 (2023): e2138–e2150. 37813577 10.1212/WNL.0000000000207866 PMC10663033

[17] A. Liberati , D. G. Altman , J. Tetzlaff , et al., “The PRISMA Statement for Reporting Systematic Reviews and Meta‐Analyses of Studies That Evaluate Health Care Interventions: Explanation and Elaboration,” Journal of Clinical Epidemiology 62 (2009): e1–e34. 19631507 10.1016/j.jclinepi.2009.06.006

[18] D. I. Friedman , G. T. Liu , and K. B. Digre , “Revised Diagnostic Criteria for the Pseudotumor Cerebri Syndrome in Adults and Children,” Neurology 81 (2013): 1159–1165. 23966248 10.1212/WNL.0b013e3182a55f17

[19] J. A. C. Sterne , J. Savović , M. J. Page , et al., “RoB 2: A Revised Tool for Assessing Risk of Bias in Randomised Trials,” BMJ 366 (2019): l4898. 31462531 10.1136/bmj.l4898

[20] J. A. Sterne , M. A. Hernán , B. C. Reeves , et al., “ROBINS‐I: A Tool for Assessing Risk of Bias in Non‐Randomised Studies of Interventions,” BMJ 355 (2016): i4919. 27733354 10.1136/bmj.i4919 PMC5062054

[21] X. Wan , W. Wang , J. Liu , and T. Tong , “Estimating the Sample Mean and Standard Deviation From the Sample Size, Median, Range and/or Interquartile Range,” BMC Medical Research Methodology 14 (2014): 135. 25524443 10.1186/1471-2288-14-135 PMC4383202

[22] M. F. Freeman and J. W. Tukey , “Transformations Related to the Angular and the Square Root,” Annals of Mathematical Statistics 21 (1950): 607–611.

[23] J. Cohen , Statistical Power Analysis for the Behavioral Sciences (Routledge, 2013).

[24] M. I. Stefanou , L. Palaiodimou , A. H. Katsanos , et al., “The Effects of HMG‐CoA Reductase Inhibitors on Disease Activity in Multiple Sclerosis: A Systematic Review and Meta‐Analysis,” Multiple Sclerosis and Related Disorders 58 (2022): 103395. 35216778 10.1016/j.msard.2021.103395

[25] J. J. Deeks , J. P. Higgins , D. G. Altman , and Group CSM , “Analysing Data and Undertaking Meta‐Analyses,” in Cochrane Handbook for Systematic Reviews of Interventions (Wiley, 2019), 241–284.

[26] L. Palaiodimou , M. I. Stefanou , A. H. Katsanos , et al., “Cerebral Venous Sinus Thrombosis and Thrombotic Events After Vector‐Based COVID‐19 Vaccines: A Systematic Review and Meta‐Analysis,” Neurology 97 (2021): e2136–e2147. 34610990 10.1212/WNL.0000000000012896

[27] N. Krajnc , B. Itariu , S. Macher , et al., “Treatment With GLP‐1 Receptor Agonists Is Associated With Significant Weight Loss and Favorable Headache Outcomes in Idiopathic Intracranial Hypertension,” Journal of Headache and Pain 24 (2023): 89. 37460968 10.1186/s10194-023-01631-z PMC10353241

[28] G. S. Sioutas , W. Mualem , J. Reavey‐Cantwell , and D. J. Rivet , “GLP‐1 Receptor Agonists in Idiopathic Intracranial Hypertension,” JAMA Neurology 82, no. 1 (2025): 887–894. 40658395 10.1001/jamaneurol.2025.2020 PMC12261113

[29] A. Y. Azzam , M. A. Essibayi , N. Farkas , et al., “Efficacy of Tirzepatide Dual GIP/GLP‐1 Receptor Agonist in Patients With Idiopathic Intracranial Hypertension. A Real‐World Propensity Score‐Matched Study,” Endocrinology, Diabetes & Metabolism 8 (2025): e70019. 10.1002/edm2.70019 PMC11825589 39949069

[30] S. P. Mollan , J. Hoffmann , and A. J. Sinclair , “Advances in the Understanding of Headache in Idiopathic Intracranial Hypertension,” Current Opinion in Neurology 32 (2019): 92–98. 30547900 10.1097/WCO.0000000000000651 PMC6343949

[31] S. Tepper , M. Ashina , U. Reuter , et al., “Safety and Efficacy of Erenumab for Preventive Treatment of Chronic Migraine: A Randomised, Double‐Blind, Placebo‐Controlled Phase 2 Trial,” Lancet Neurology 16 (2017): 425–434. 28460892 10.1016/S1474-4422(17)30083-2

[32] J. L. Mitchell , S. P. Mollan , V. Vijay , and A. J. Sinclair , “Novel Advances in Monitoring and Therapeutic Approaches in Idiopathic Intracranial Hypertension,” Current Opinion in Neurology 32 (2019): 422–431. 30865008 10.1097/WCO.0000000000000690 PMC6522204

[33] O. Grech , J. L. Mitchell , H. S. Lyons , et al., “Effect of Glucagon Like Peptide‐1 Receptor Agonist Exenatide, Used as an Intracranial Pressure Lowering Agent, on Cognition in Idiopathic Intracranial Hypertension,” Eye 38 (2024): 1374–1379. 38212401 10.1038/s41433-023-02908-y PMC11076535

[34] J. L. Mitchell , H. S. Lyons , J. K. Walker , et al., “A Randomized Sequential Cross‐Over Trial Evaluating Five Purportedly ICP‐Lowering Drugs in Idiopathic Intracranial Hypertension,” Headache 65 (2025): 258–268. 39853738 10.1111/head.14897 PMC11794974

[35] S. Luan , W. Cheng , C. Wang , J. Gong , and J. Zhou , “Impact of Glucagon‐Like Peptide 1 Analogs on Cognitive Function Among Patients With Type 2 Diabetes Mellitus: A Systematic Review and Meta‐Analysis,” Frontiers in Endocrinology 13 (2022): 1047883. 36387915 10.3389/fendo.2022.1047883 PMC9650490

[36] M. Wall , M. P. McDermott , K. D. Kieburtz , et al., “Effect of Acetazolamide on Visual Function in Patients With Idiopathic Intracranial Hypertension and Mild Visual Loss: The Idiopathic Intracranial Hypertension Treatment Trial,” Journal of the American Medical Association 311 (2014): 1641–1651. 24756514 10.1001/jama.2014.3312 PMC4362615

[37] M. Thaller , V. Homer , Y. Hyder , et al., “The Idiopathic Intracranial Hypertension Prospective Cohort Study: Evaluation of Prognostic Factors and Outcomes,” Journal of Neurology 270 (2023): 851–863. 36242625 10.1007/s00415-022-11402-6 PMC9886634

[38] S. Siriyotha , T. Anothaisintawee , P. Looareesuwan , et al., “Effectiveness of Glucagon‐Like Peptide‐1 Receptor Agonists for Reduction of Body Mass Index and Blood Glucose Control in Patients With Type 2 Diabetes Mellitus and Obesity: A Retrospective Cohort Study and Difference‐In‐Difference Analysis,” BMJ Open 14 (2024): e086424. 10.1136/bmjopen-2024-086424 PMC11590856 39581734

[39] M. I. Stefanou , A. Theodorou , K. Malhotra , et al., “Risk of Major Adverse Cardiovascular Events and Stroke Associated With Treatment With GLP‐1 or the Dual GIP/GLP‐1 Receptor Agonist Tirzepatide for Type 2 Diabetes: A Systematic Review and Meta‐Analysis,” European Stroke Journal 9 (2024): 530–539. 38400569 10.1177/23969873241234238 PMC11418422

[40] W. Halloum , Y. A. Dughem , D. Beier , and L. Pellesi , “Glucagon‐Like Peptide‐1 (GLP‐1) Receptor Agonists for Headache and Pain Disorders: A Systematic Review,” Journal of Headache and Pain 25 (2024): 112. 38997662 10.1186/s10194-024-01821-3 PMC11241973

[41] E. Bakola , D. Alonistiotis , C. Arvaniti , et al., “Optic Disc Drusen Mimicking Idiopathic Intracranial Hypertension (IIH): Rely on Ultrasound,” Neurological Research and Practice 3 (2021): 33. 34120652 10.1186/s42466-021-00133-0 PMC8201862

[42] E. Bakola , L. Palaiodimou , A. Eleftheriou , et al., “Transorbital Sonography in Idiopathic Intracranial Hypertension: Single‐Center Study, Systematic Review and Meta‐Analysis,” Journal of Neuroimaging 34 (2024): 108–119. 37822030 10.1111/jon.13160

[43] R. J. Piper , A. V. Kalyvas , A. M. Young , M. A. Hughes , A. A. Jamjoom , and I. P. Fouyas , “Interventions for Idiopathic Intracranial Hypertension,” Cochrane Database of Systematic Reviews 2015 (2015): Cd003434. 26250102 10.1002/14651858.CD003434.pub3 PMC7173709

[44] National Library of Medicine , “A Trial to Determine the Efficacy and Safety of Presendin in IIH (IIH EVOLVE), ClinicalTrials.Gov Identifier: NCT05347147,” (2024), https://clinicaltrials.gov/study/NCT05347147.

[45] A. Yiangou , J. L. Mitchell , M. Nicholls , et al., “Obstructive Sleep Apnoea in Women With Idiopathic Intracranial Hypertension: A Sub‐Study of the Idiopathic Intracranial Hypertension Weight Randomised Controlled Trial (IIH: WT),” Journal of Neurology 269 (2022): 1945–1956. 34420064 10.1007/s00415-021-10700-9 PMC8940816

