# Supplementary material for: Efficacy and Safety of GLP‐1 and Dual GIP/GLP‐1 Receptor Agonists in Idiopathic Intracranial Hypertension: A Systematic Review and Meta‐Analysis
Source: Eur J Neurol. 2025 Sep 12;32(9):e70358. doi: 10.1111/ene.70358 (PMC12426903; doi:10.1111/ene.70358)
Supplement: Supplementary file 1 — Data S1: Supporting Information. [file ENE-32-e70358-s001.docx]

**SUPPLEMENTAL MATERIAL**

**Efficacy and Safety of GLP-1 and Dual GIP/GLP-1 Receptor Agonists in Idiopathic Intracranial Hypertension: a systematic review and meta-analysis**

Maria-Ioanna Stefanou, MD^1,2^, Irini Chatziralli, MD^3^, Vaia Lambadiari, MD^4^ Annerose Mengel, MD^2,5^, Aikaterini Foska, MD^1^, Maria Chondrogianni, MD^1^, Eleni Bakola, MD^1^, Panagiota-Eleni Tsalouchidou, MD^1^, Dimos D Mitsikostas, MD^6^, Gerasimos Siasos, MD^7^, Ulf Ziemann, MD^2,5^, Georgios Tsivgoulis, MD^1^*

*^1^Second Department of Neurology, “Attikon” University Hospital, School of Medicine, National and Kapodistrian University of Athens, Athens, Greece.*

*^2^Department of Neurology & Stroke, Eberhard-Karls University of Tübingen, Tübingen, Germany.*

*^3^Second Department of Ophthalmology, Attikon University Hospital, National and Kapodistrian University of Athens, Athens, Greece.*

*^4^Second Department of Internal Medicine, “Attikon” University Hospital, School of Medicine, National and Kapodistrian University of Athens, Athens, Greece.*

*^5^Hertie Institute for Clinical Brain Research, Eberhard-Karls University of Tübingen, Tübingen, Germany.*

*^6^First Neurology Department, Aeginition Hospital, Medical School, National and Kapodistrian University of Athens, Athens, Greece.*

*^7^Third Department of Cardiology, Sotiria Thoracic Diseases General Hospital, National and Kapodistrian University of Athens, Athens, Greece.*

**Correspondence**

Dr. Georgios Tsivgoulis

Second Department of Neurology, “Attikon” University Hospital, School of Medicine, National and Kapodistrian University of Athens, Greece

Rimini 1, Chaidari, Athens, Greece 12462

Email: [tsivgoulisgiorg@yahoo.gr](mailto:tsivgoulisgiorg@yahoo.gr)

Tel: +30 6937178635; Fax: +30 2105832471

**Complete search algorithm used in MEDLINE search.**

**Complete search algorithm used in SCOPUS search.**

**Supplementary Tables:**

Supplemental Table-S1. Table of excluded studies with reasons for exclusion.

**Supplementary Figures:**

Supplementary Figure-S1. Flowchart presenting the selection of eligible studies.

Supplementary Figure-S2. Traffic Light Plot presenting the quality assessment of included RCTs using the risk of bias in randomized trials (RoB 2) tool.

Supplementary Figure-S3. Traffic Light Plot presenting the quality assessment of included non-randomized trials using the ROBINS-I tool.

Supplementary Figure-S4: Forest plot comparing the change in body mass index at 3 months in IIH patients treated with GLP-1RAs versus controls.

Supplementary Figure-S5: Forest plot comparing the change in body mass index at the end of follow-up in IIH patients treated with GLP-1RAs versus controls.

Supplementary Figure-S6: Forest plot comparing the change in the visual field of the most affected eye at 3 months in IIH patients treated with GLP-1RAs versus controls.

Supplementary Figure-S7: Forest plot comparing the change in the visual field of the most affected eye at the end of follow-up in IIH patients treated with GLP-1RAs versus controls.

Supplementary Figure0S8: Forest plot comparing the change RNFL thickness of the most affected eye at 3 months in IIH patients treated with GLP-1RAs versus controls.

Supplementary Figure-S9: Forest plot comparing the change RNFL thickness of the most affected eye at the end of follow-up in IIH patients treated with GLP-1RAs versus controls.

Supplementary Figure-S10: Forest plot of the pooled incidence of SAEs among IIH patients treated with GLP-1 RAs.

Supplementary Figure-S11: Forest plot of the pooled incidence of AEs leading to premature discontinuation of GLP-1 RAs.

Supplementary Figure-S12: Forest plot of the pooled incidence of mild gastrointestinal AEs among IIH patients treated with GLP-1 RAs.

Supplementary Figure-S13: Forest plot of the pooled incidence of nausea among IIH patients treated with GLP-1 RAs.

**ANALYSIS**

**Expanded Methods**

**Complete search algorithm used in MEDLINE search**

(GLP-1 receptor agonists OR GLP-1 OR GLP-1 RA OR GLP1RA OR GIP/GLP-1 OR lixisenatide OR exenatide OR liraglutide OR semaglutide OR albiglutide OR dulaglutide OR tirzepatide) AND (idiopathic intracranial hypertension OR pseudotumor cerebri)

**Complete search algorithm used in SCOPUS search**

TITLE-ABS-KEY ( {GLP-1} OR {GLP-1 receptor agonists} OR {GLP-1 RA} OR {GLP1RA} OR { GIP/GLP-1} OR {lixisenatide} OR {exenatide} OR {liraglutide} OR {semaglutide} OR {albiglutide} OR {dulaglutide} OR {tirzepatide}) AND TITLE-ABS-KEY ( {idiopathic intracranial hypertension} OR {pseudotumor cerebri})

**Supplemental Tables**

**Supplemental Table-S1.** Table of excluded studies with reasons for exclusion.

| **PMID** | **Reason for exclusion** |
| --- | --- |
| 37948151 | Case report |
| 37901040 | Out-of-scope |
| 38212401 | Overlapping populations |
| 39949066 | Overlapping populations |
| 40344770 | Overlapping populations |
| 40301667 | Study design (non-matched with significant baseline differences between groups; in addition outcomes of interest not aligned with our predefined endpoints) |
| 28835515 | Preclinical |
| 39677436 | Preprint |
| 39677446 | Preprint |
| 29985799 | Review |
| 30865008 | Review |
| 31767019 | Review |
| 32700012 | Review |
| 33200788 | Review |

**Supplemental Figures**

**Figure S1:** Flowchart presenting the selection of eligible studies.


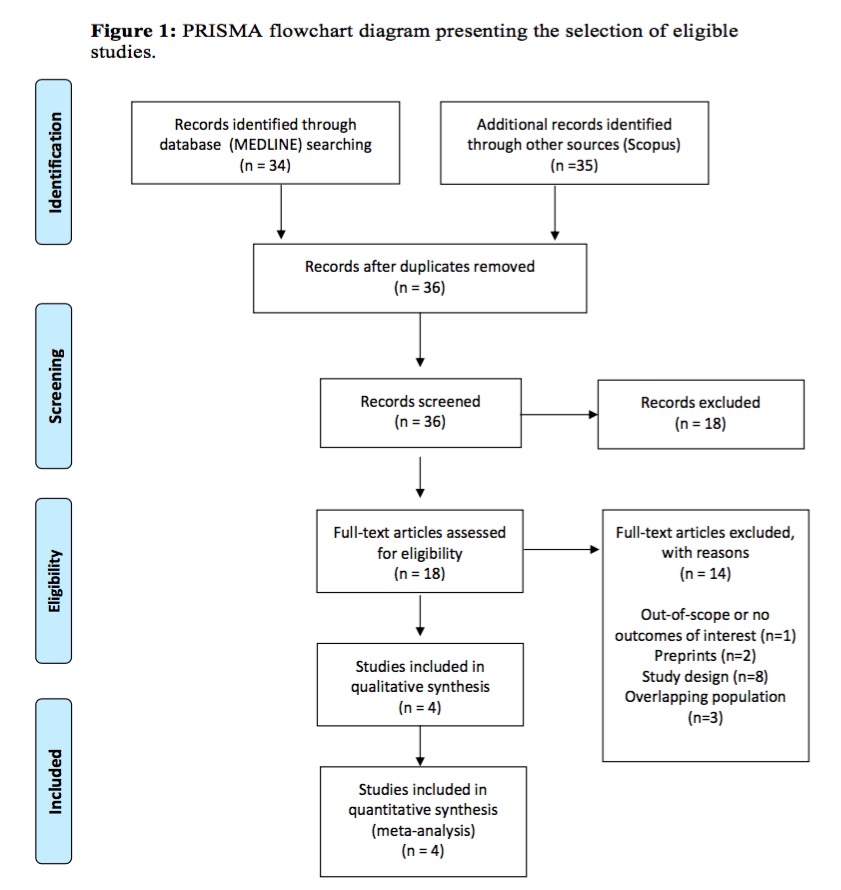


**Figure S2.** Traffic Light Plot presenting the quality assessment of included RCTs using the risk of bias in randomized trials (RoB 2) tool.

The risk of bias in Mitchell et al.^1^ was assessed using the RoB 2 tool^2^. Selection bias was low due to computer-generated randomization and well-balanced baseline characteristics. Performance bias was minimized through double-blinding. Attrition bias was low, as missing data were minimal, with only one telemetric failure. Measurement bias was moderate, as a higher statistical threshold (p < 0.1) was applied, increasing the risk of false positives. Reporting bias was low, as pre-specified outcomes were reported. Potential funding bias raises concerns about reporting emphasis, contributing to an overall low to moderate risk of bias.


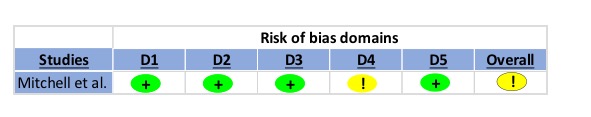

**Figure S3.**Traffic Light Plot presenting the quality assessment of included non-randomized trials or registry studies using the ROBINS-I tool.

The risk of bias in Krajnc et al.^3^, Azzam et al.^4^ and Sioutas et al.^5^ was assessed using the ROBINS-I tool^6^. All studies were observational, carrying inherent risk of residual confounding. Sioutas et al. and Azzam et al. (TriNetX retrospective cohorts) had serious risk from unmeasured confounding and participant selection; Krajnc et al. (single-center open-label) had critical confounding and selection bias due to complete self-allocation. Intervention classification was low risk for the registry-based studies and moderate for Krajnc et al. due to open-label awareness. Deviations from intended interventions were serious for Sioutas et al., moderate for Azzam et al., and serious for Krajnc et al. Missing data risk was moderate for Sioutas et al., low for Azzam et al. (minimal, balanced attrition), and low for Krajnc et al. Outcome measurement was moderate for all due to reliance on self-reported headache improvement. Selective reporting risk was moderate for Sioutas et al. and Azzam et al., and serious for Krajnc et al. Overall risk was serious for Sioutas et al. and Azzam et al., and moderate-to-serious for Krajnc et al.

**
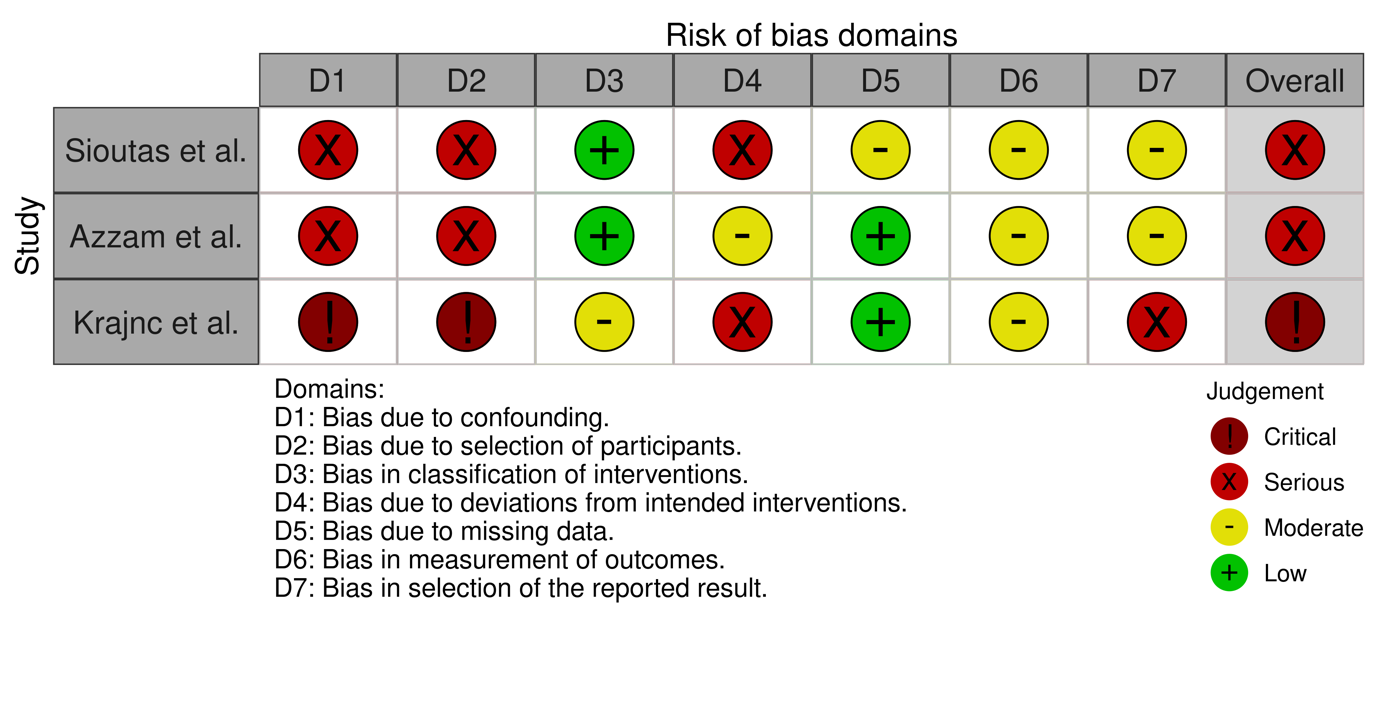
**

**
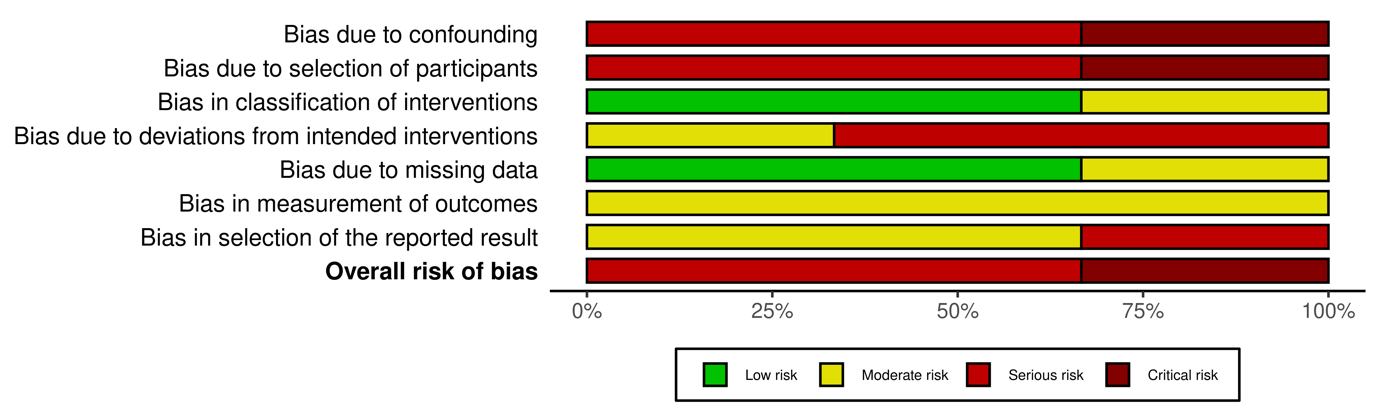
**

**Figure S4**: Forest plot comparing the change in body mass index at 3 months in IIH patients treated with GLP-1RAs versus controls.

**
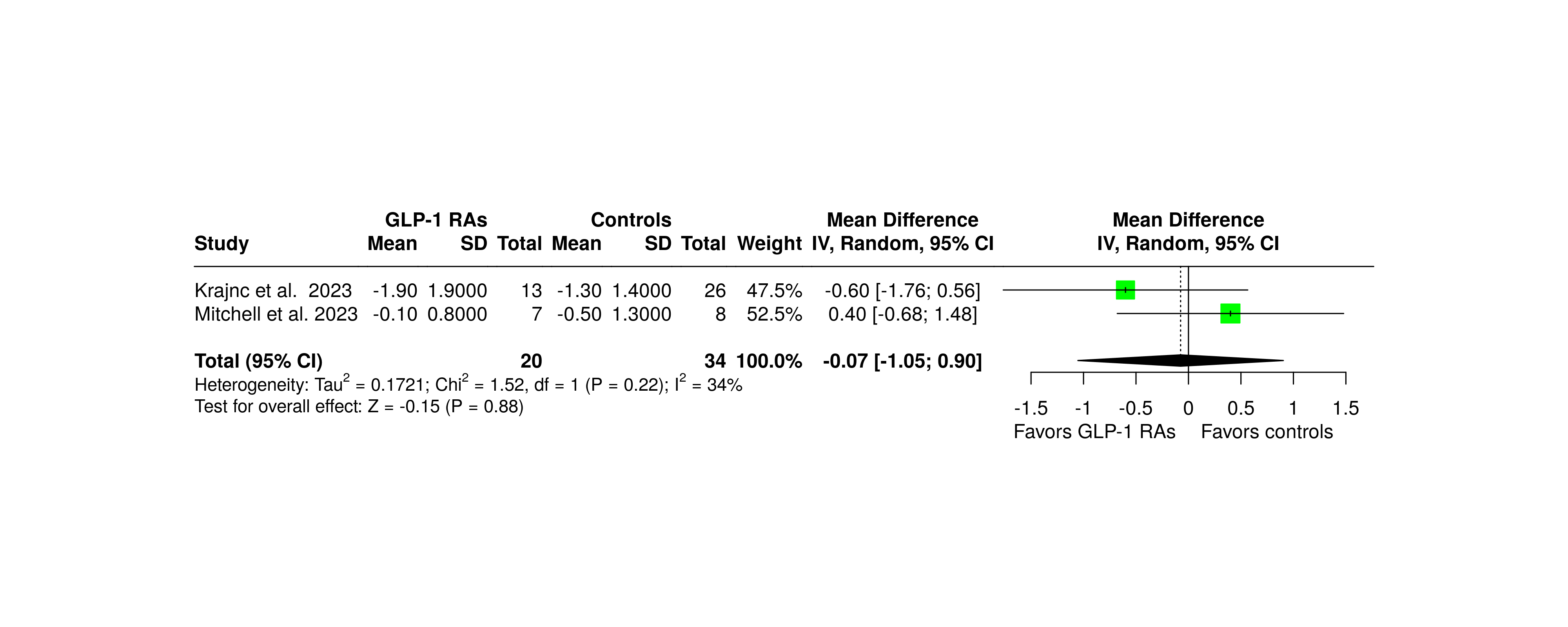
**

**Figure S5**: Forest plot comparing the change in body mass index at the end of follow-up in IIH patients treated with GLP-1RAs versus controls.

**
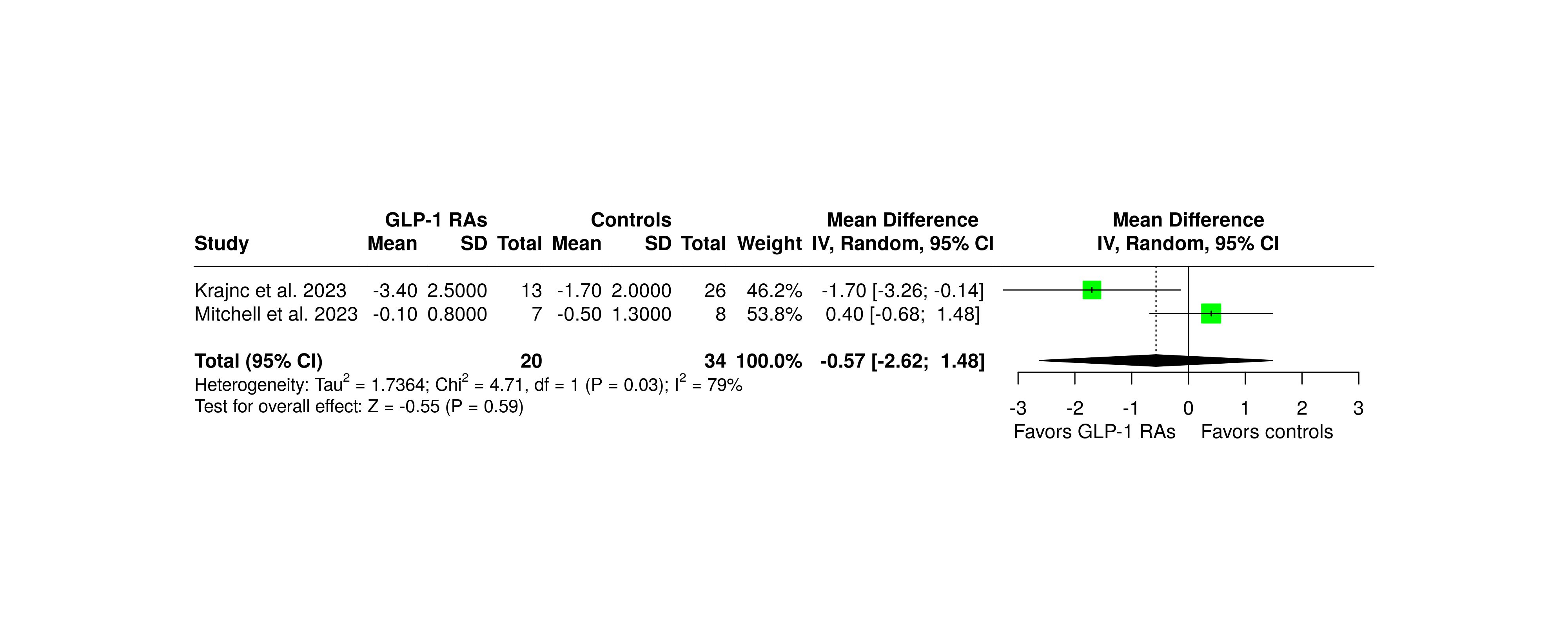
**

**Figure S6**: Forest plot comparing the change in the visual field of the most affected eye at 3 months in IIH patients treated with GLP-1RAs versus controls.

**
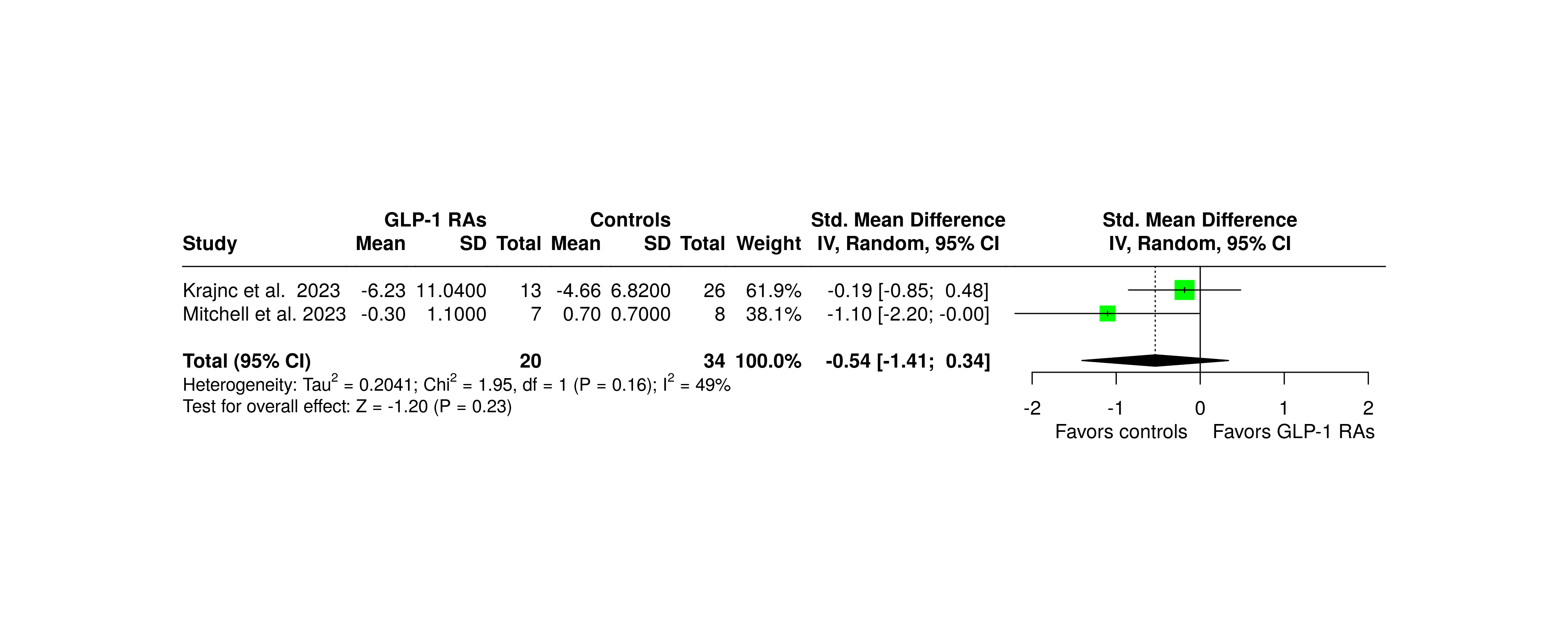
**

**Figure S7**: Forest plot comparing the change in the visual field of the most affected eye at the end of follow-up in IIH patients treated with GLP-1RAs versus controls.

**
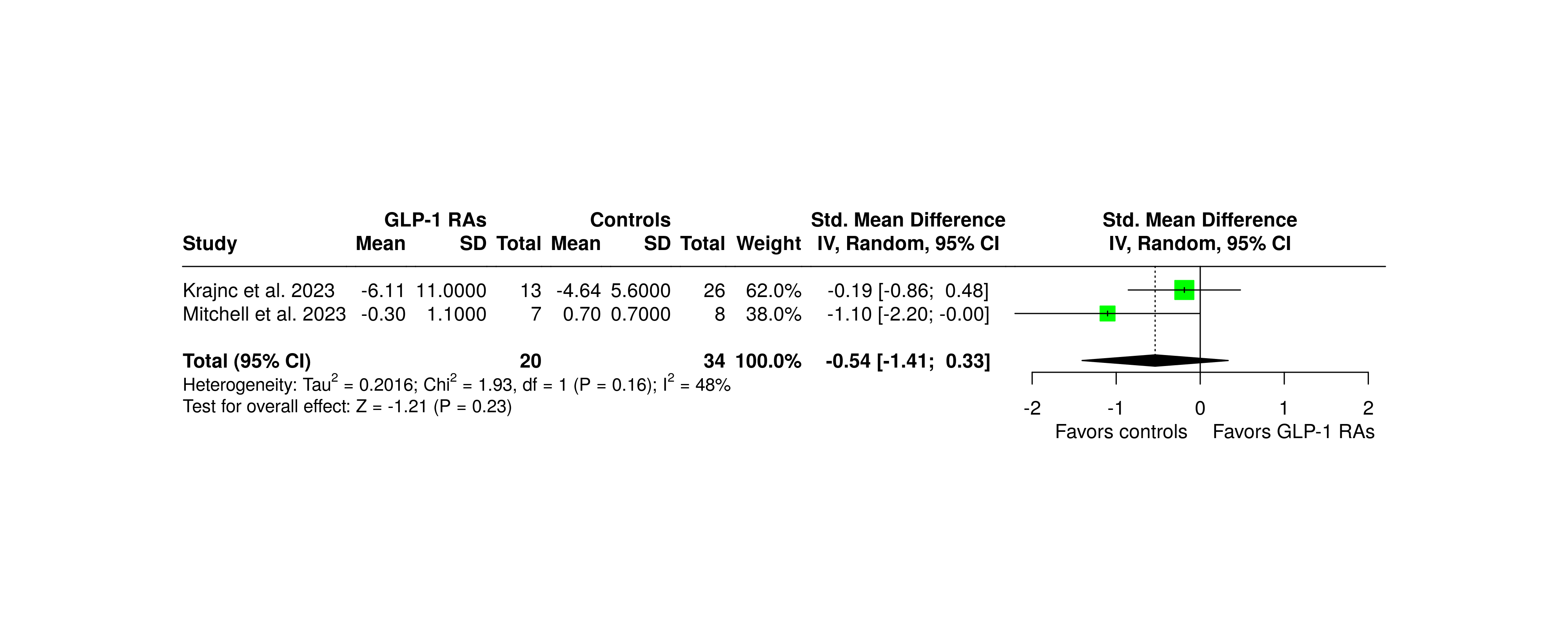
**

**Figure S8**: Forest plot comparing the change RNFL thickness of the most affected eye at 3 months in IIH patients treated with GLP-1RAs versus controls.

**
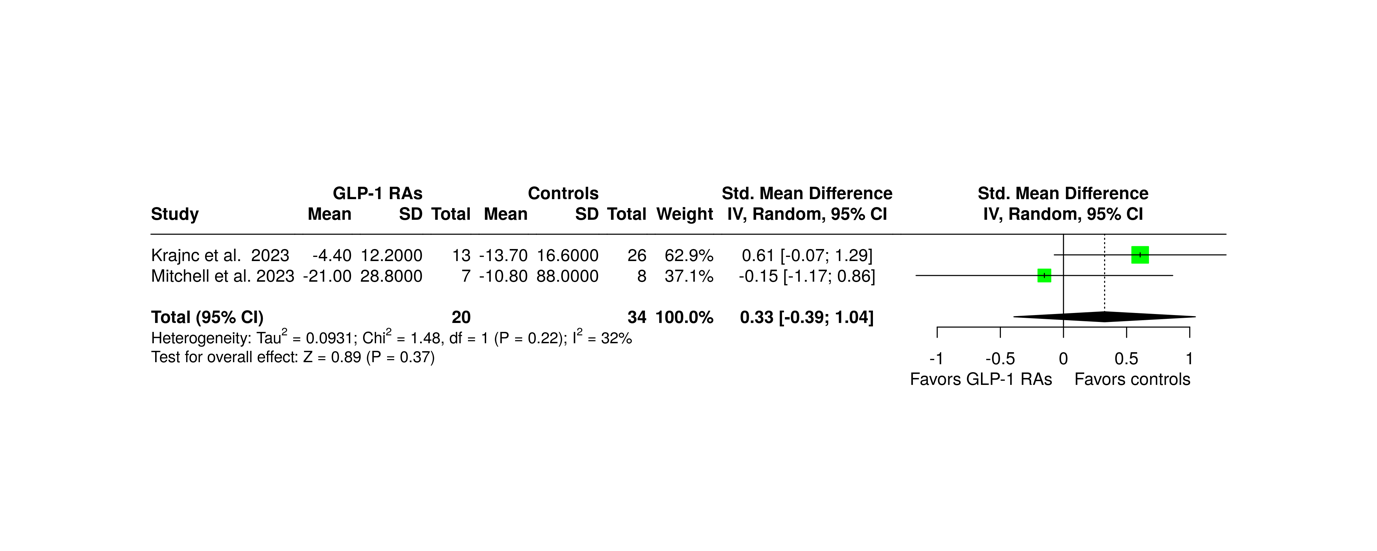
**

**Figure S9**: Forest plot comparing the change RNFL thickness of the most affected eye at the end of follow-up in IIH patients treated with GLP-1RAs versus controls.

**
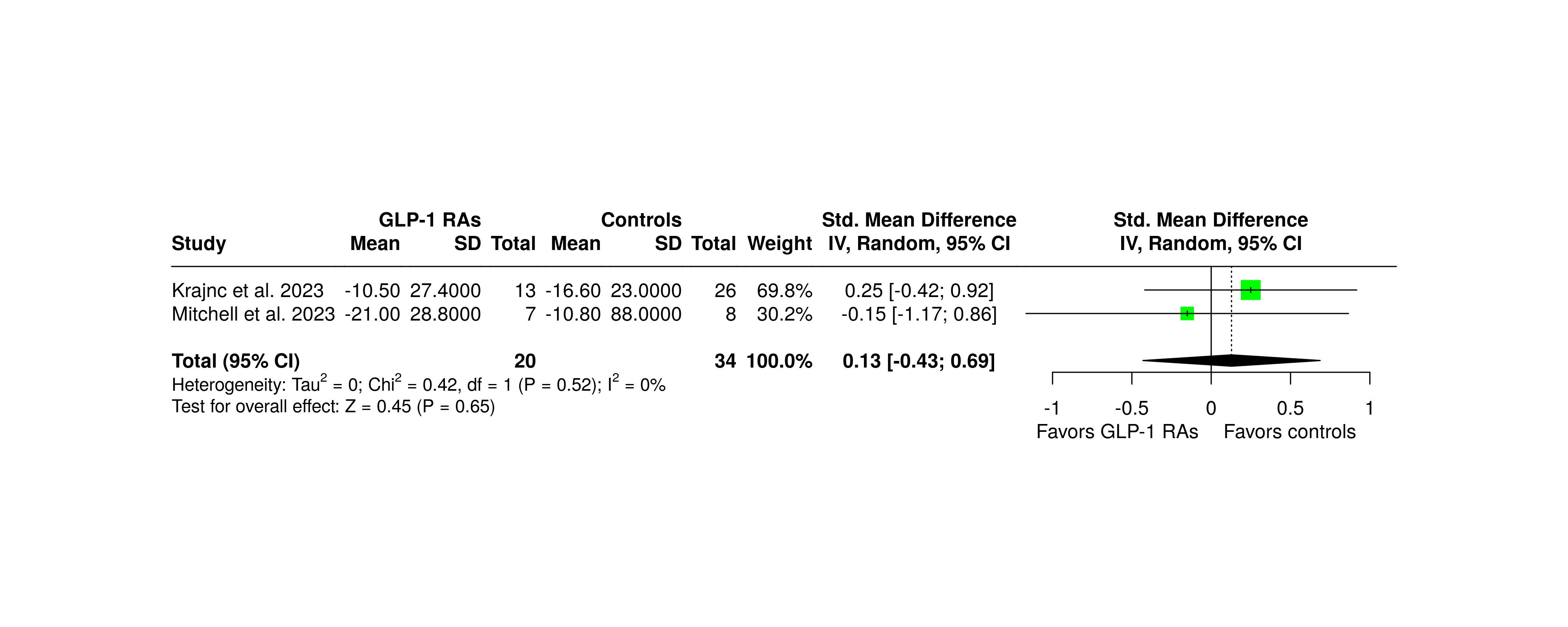
**

**Figure S10**: Forest plot of the pooled incidence of SAEs among IIH patients treated with GLP-1 RAs. Note that zero events were recorded for each of the aforementioned safety outcomes (continuity correction applied).

**
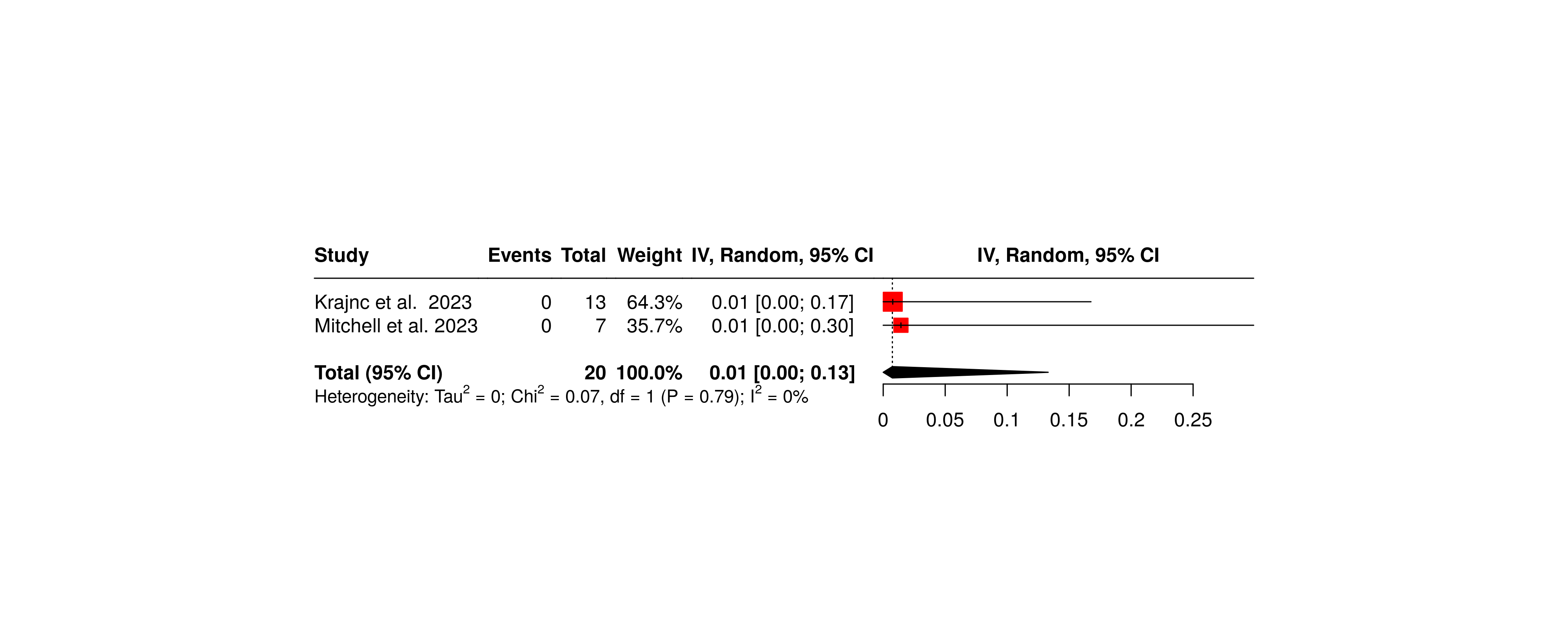
**

**Figure S11**: Forest plot of the pooled incidence of AEs leading to premature discontinuation of GLP-1 RAs. Note that zero events were recorded for each of the aforementioned safety outcomes (continuity correction applied).

**
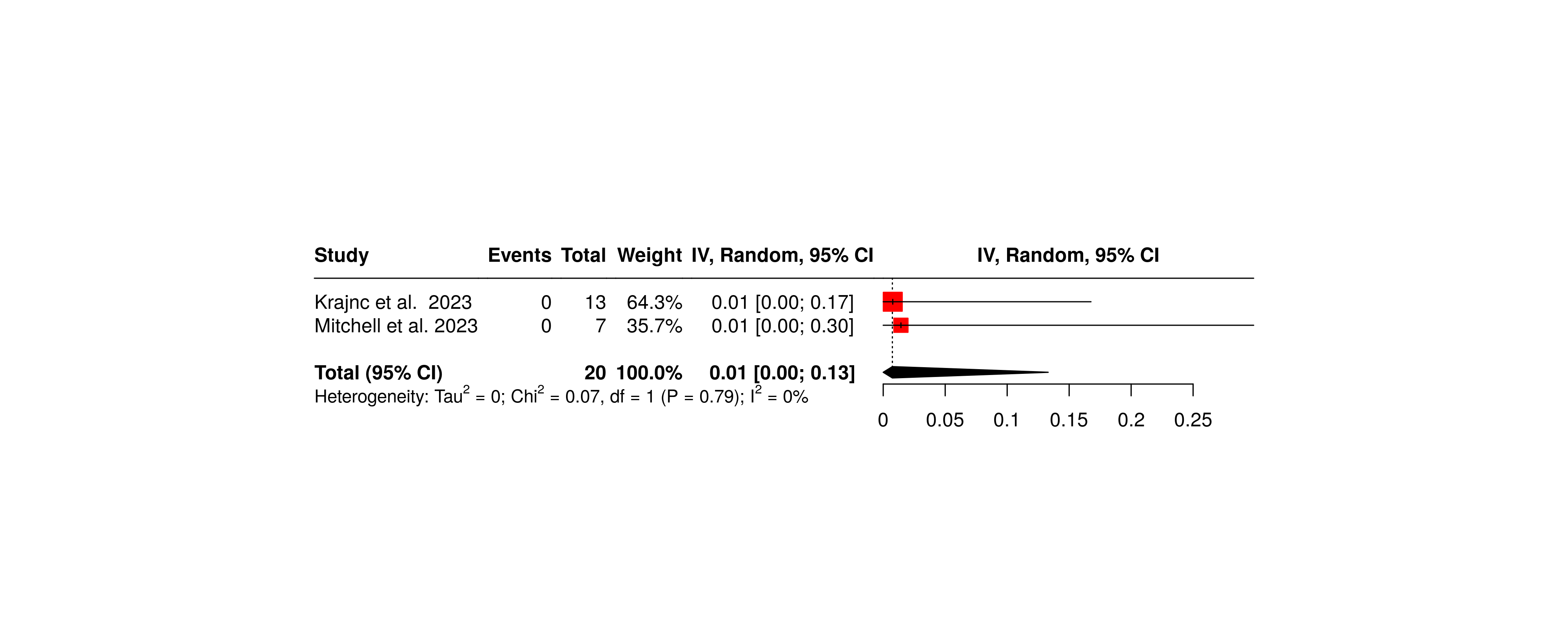
**

**Figure S12**: Forest plot of the pooled incidence of mild gastrointestinal AEs among IIH patients treated with GLP-1 RAs.

**
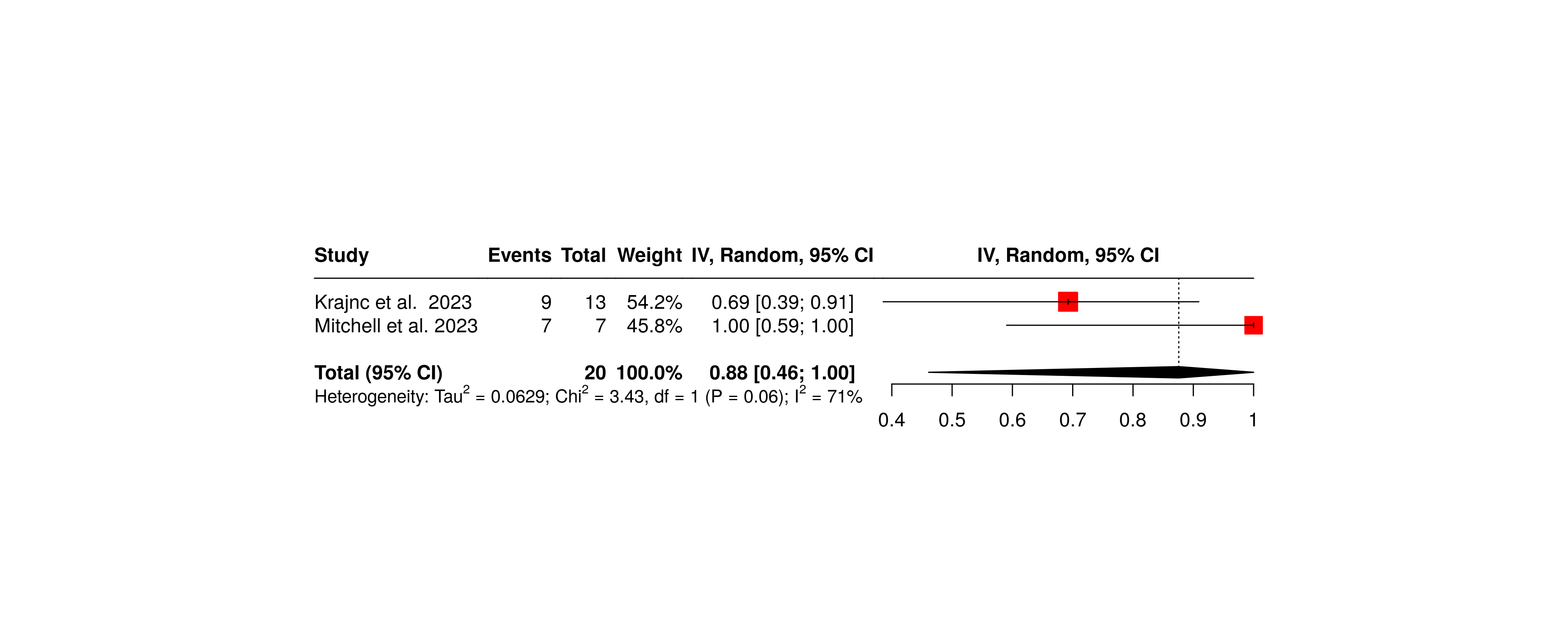
**

**Figure S13**: Forest plot of the pooled incidence of nausea among IIH patients treated with GLP-1 RAs.

**
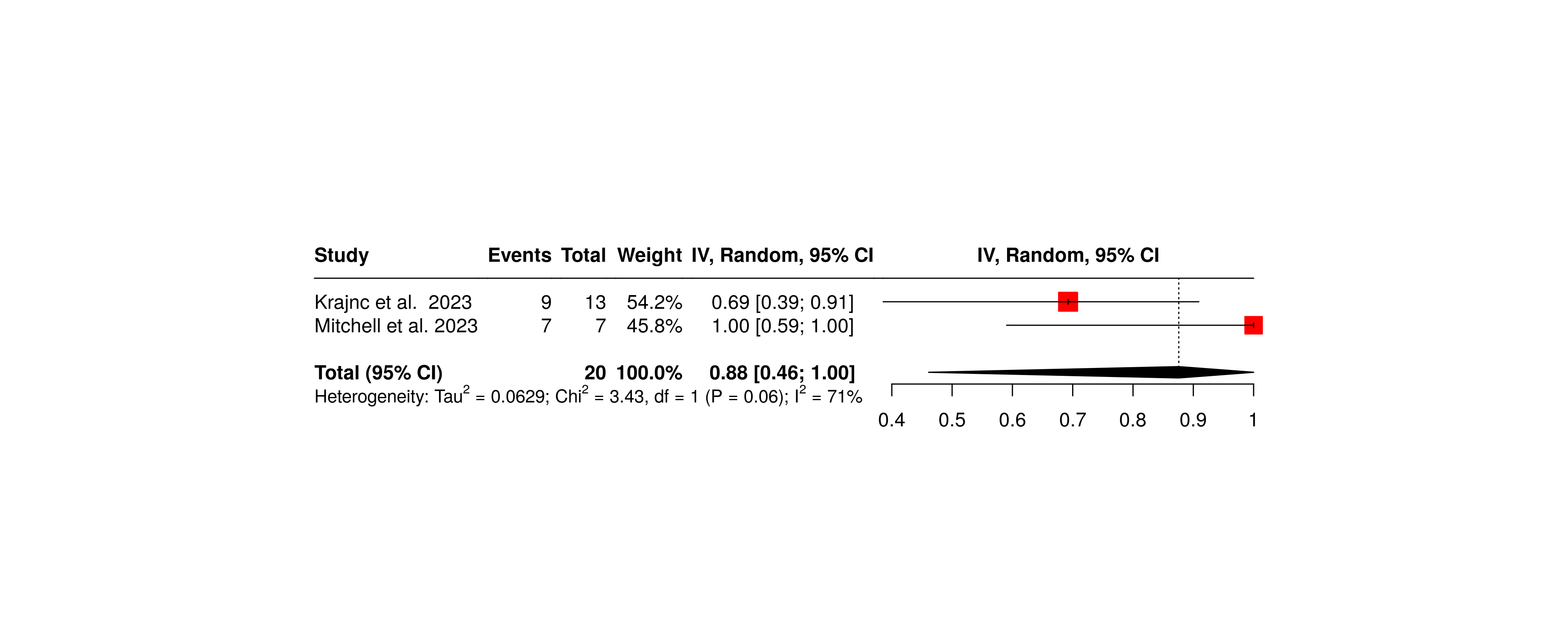
**

**References:**

1. Mitchell JL, Lyons HS, Walker JK, et al. The effect of GLP-1RA exenatide on idiopathic intracranial hypertension: a randomized clinical trial. *Brain* 2023;146(5):1821-30. doi: 10.1093/brain/awad003

2. Sterne JAC, Savović J, Page MJ, et al. RoB 2: a revised tool for assessing risk of bias in randomised trials. *Bmj* 2019;366:l4898. doi: 10.1136/bmj.l4898

3. Krajnc N, Itariu B, Macher S, et al. Treatment with GLP-1 receptor agonists is associated with significant weight loss and favorable headache outcomes in idiopathic intracranial hypertension. *J Headache Pain* 2023;24(1):89. doi: 10.1186/s10194-023-01631-z

4. Azzam AY, Essibayi MA, Farkas N, Azab MA, Morsy MM, Elamin O, et al. Efficacy of Tirzepatide Dual GIP/GLP-1 Receptor Agonist in Patients With Idiopathic Intracranial Hypertension. A Real-World Propensity Score-Matched Study. Endocrinol Diabetes Metab. 2025 8: e70019.

5. Sioutas GS, Mualem W, Reavey-Cantwell J, Rivet DJ, 2nd. GLP-1 Receptor Agonists in Idiopathic Intracranial Hypertension. JAMA Neurol. 2025.

6. Sterne JA, Hernán MA, Reeves BC, et al. ROBINS-I: a tool for assessing risk of bias in non-randomised studies of interventions. *Bmj* 2016;355:i4919. doi: 10.1136/bmj.i4919
